# Etymologia

**DOI:** 10.3201/eidET170.ET1710

**Published:** 2011-10

**Affiliations:** Edited by Nancy Männikkö; email: nmannikko@cdc.gov

**Keywords:** Plasmodium knowlesi, malaria, Robert Knowles, etymologia

## *Plasmodium knowlesi* [plaz-mo’de-əm no-le-se]

From the Greek, *plasma*, anything formed or molded, and Robert Knowles, a physician with the Indian Medical Service, credited with discovery of the organism. Knowles himself attributed the finding of *P. knowlesi* to 2 colleagues (L.E. Napier and H.G.M. Campbell) at the School of Tropical Medicine and Hygiene in Calcutta who found the protozoan while investigating kala-azar transmission. “Knowing that we should be interested in the strain from a protozoological point of view,” Knowles wrote, “[Napier] handed over the original monkey to my assistant, Dr. B.M. Das Gupta.” Das Gupta maintained the strain in monkeys until he and Knowles were able to carry out human infection experiments, which they reported in 1932.Sources: Centers for Disease Control and Prevention. The history of malaria, an ancient disease [cited 2011 Jul 22]. http://www.cdc.gov/malaria/about/history; Dorland’s illustrated medical dictionary. 31st ed. Philadelphia: Saunders; 2007; Knowles R. Monkey malaria. BMJ. 1935;2:1020. doi:10.1136/bmj.2.3907.1020; Knowles R, Das Gupta BM. A study of monkey-malaria, and its experimental transmission to man. Ind Med Gaz. 1932;67:301–21.

